# Mutations Encountered in Acute Lymphoblastic Leukemia: A Retrospective Study in a Teaching Hospital in Jeddah, Saudi Arabia

**DOI:** 10.7759/cureus.12426

**Published:** 2021-01-02

**Authors:** Mohamad H Qari, Ali Alawi Alattas, Sultan Mohammed Binkuddah, Abdullah K Almarri, Suhayb Shafy, Salem Khalifah Alsulami, Jumana Alzuhayri

**Affiliations:** 1 Internal Medicine, King Abdulaziz University, Jeddah, SAU; 2 Medicine, King Abdulaziz University, Jeddah, SAU; 3 Internal Medicine, Ibn Sina National College, Jeddah, SAU; 4 Medicine, Jeddah University, Jeddah, SAU; 5 Biochemistry, King Abdulaziz University, Jeddah, SAU

**Keywords:** mutations, acute lymphoblastic leukemia (all)

## Abstract

Background

Acute lymphoblastic leukemia (ALL) is an invasive cancer that results from the malignant conversion and rapid replication of white blood cells and hematopoietic stem cells that supply multiple lymphocytes. Harmful gene mutations occur in more than two-thirds of patients with ALL; however, these mutations have not been extensively identified in Saudi Arabia.

Aim

The aim of this study was to identify the types of mutations in patients with ALL at King Abdulaziz University Hospital (KAUH) in Jeddah. In addition, we identified the most common mutations.

Methods

A retrospective study was performed on patients who were diagnosed with ALL from January 2009 to January 2019 at the Department of Hematology at KAUH. Our target population comprised patients diagnosed with ALL, including all age groups and both sexes. Patients were excluded if they had Down syndrome or central nervous system involvement, Li-Fraumeni syndrome, or neurofibromatosis.

Results

Of the 130 patients with ALL, 101 (77.77%) were children. The number of men (n=81) was substantially more than that of women (n=49). The data showed that 13.1% of our patients had mutations, and they occurred more frequently in patients with B-cell lymphoblastic ALL (B-ALL) than in those with T-cell lymphoblastic ALL (T-ALL). Several mutations, including BCR-ABL and ETV6/RUNX1, were more common in B-ALL, whereas the MLL-F0X04mutation was more commonly observed in T-ALL. There was a significant difference between the types of ALL and the genes involved (p=0.039). One female patient had translocation t(X;11)(q26;q23) (MLL-F0X04), which is a rare mutation.

Conclusion

In summary, 13.1% of our study population had mutations. The BCR-ABL fusion gene was the most frequent mutation in patients at KAUH, and it occurred at a higher rate in B-ALL. Moreover, we detected other mutations, such as ETV6/RUNX1 and MLL-F0X04. The gene mutations were significantly different between B-ALL and T-ALL.

## Introduction

Acute lymphoblastic leukemia (ALL) is a hematological cancer that is related to the abnormal replication of white blood cells (WBCs) in the bone marrow, leading to the immature generation of lymphocytes [1]. Both adults and children can be affected. ALL is classified into B-cell lymphoblastic (B-ALL) and T-cell lymphoblastic (T-ALL) according to the World Health Organization [2], with B-ALL being the most common type [3]. Conversions of these cells will lead to altered blast cell proliferation, survival, and maturation, and eventually to the lethal accumulation of leukemic cells [2,4]. ALL is the most common malignancy in childhood, representing 25% of all childhood cancers, particularly in children between the ages of two and five years [5,6].

Approximately 20-25% of children with ALL will experience a relapse of the disease that is considered to be a major cause of treatment failure in ALL [7]. According to the American Cancer Society, 80% of ALL cases occur in children. There has been a significant improvement in the therapy of ALL; at present, the five-year overall survival rate exceeds 85% in pediatric patients; however, it remains below 45% in adults [8].

More than two-thirds of ALL cases harbor harmful mutations, including deletion, translocation, and rearrangement of genes. Recently, some studies have shown that multiple mutations are associated with ALL, such as those in IKZF1, BCL-2, ABL1, and ABL2, and ETV6/RUNX1 [4,9,10]. Previous studies noted an association between the mutation and sex, age, and type of ALL. In addition, clinical characteristics, such as WBC count, are abnormal in some cases with mutations. A previous study has shown that the most common type of mutation in patients with ALL is the deletion mutation in the IKZF1 gene encoding the transcription factor IKAROS. IKAROS is associated with poor outcomes in B-cell progenitors and poor treatment responses [11]. Some studies have found that certain types of mutations occur in specific age groups, e.g., the mutation in ABL2 is more common in adults than in children, and the incidence increases with age [10]. On the other hand, a previous study showed that ETV6/RUNX1 is one of the major genetic mutations that occur in 25% of pediatric B-ALL cases [12].

Mutations associated with ALL have not been fully identified in Saudi Arabia; therefore, the aim of this study was to determine the types of mutations in patients with ALL at King Abdulaziz University Hospital (KAUH) in Jeddah. In addition, we identified the most common types of mutations in our patient population.

## Materials and methods

This was a retrospective record review non-interventional study to detect the gene mutations in patients who were diagnosed with ALL from January 2009 to January 2019 at the Department of Hematology at KAUH. The clinical outcomes of these patients were analyzed. Our study lasted for three months from January to March 2019. All study participants provided informed consent, and the study design was approved by the Unit of Biomedical Ethics at KAUH (Reference No. 600-18).

Our target population consisted of both adult and pediatric patients diagnosed with ALL. Patients were excluded if they had Down syndrome or central nervous system involvement, Li-Fraumeni syndrome, or neurofibromatosis type 1. Medical data were collected from their records in the hospital database, using Microsoft Office Excel software (Microsoft Corp., Redmond, WA, USA). The collection data sheet included age, sex, subtype of ALL, time of diagnosis of ALL, type of mutations detected by polymerase chain reaction, chemotherapy, outcomes, and relapse.

The primary predefined analysis was performed using the Statistical Package for Social Sciences (SPSS; IBM Corp., Armonk, NY, USA), and the results are presented as frequencies, percentages, means, and standard deviations. The chi-square test was used to verify the differences between variables.

## Results

A total of 130 patients with ALL were included; 101 (77.77%) were children (<14 years, 59 male and 42 female), 12 (9.2%) were adolescents (14-20 years, eight male and four female), 16 (2.3%) were adults (21-65 years, 13 male and three female), and one male patient >65 years. The number of men was substantially more than that of women (n=81 and 49, respectively). Forty-two patients experienced relapse, and we did not observe any significant differences between age and the occurrence of relapse (Table 1). Symptoms at diagnosis are shown in Table 2. 

**Table 1:** Demographic characteristics of the patients SD, standard deviation; WBCs, white blood cells; ALL, acute lymphoblastic leukemia

Characteristic	Total	Children (2 to <14 years)	Adolescents (14 to <21 years)	Adults (21 to <65 years)	≥65 years	P-value
Number		101 (77.7%)	12 (9.2%)	16 (12.3%)	1 (0.8%)	
Age						
Mean ± SD	36.35±22.69	7.16±3.644	18.08±1.50	35.187±10.27	72±0	
Range	73	12	4	37	0	
Minimum	3	2	16	22	72	
Maximum	76	14	20	59	72	
Sex						
Male	81 (62.3%)	59 (58.41%)	8 (66.66%)	13 (81.25%)	1 (100%)	0.284
Female	49 (37.7%)	42 (41.58%)	4 (33.33%)	3 (18.75%)	0	
Nationality						
Saudi	23 (17.7%)	23 (22.72%)	0	0	0	0.042
Non-Saudi	105 (82.3%)	76 (75.24%)	12 (100%)	16 (100%)	1 (100%)	
Alive	103 (79.2%)	89 (88.11%)	8 (66.66%)	6 (37.5%)	0 (%)	0.000
Died	27 (20.8%)	12 (11.88%)	4 (33.33%)	10 (62.5%)	1 (100%)	
Relapsed	42 (32.30%)	30 (29.7%)	5 (%41.66)	7 (43.75%)	0	0.526
Mutation	18 (13.8%)	12 (13.86%)	3 (25%)	3 (18.75%)	0	0.678
Level of WBCs						
High	31 (23.8%)	22 (21.78%)	4 (33.33%)	4 (25%)	1 (100%)	0.492
Normal	35 (26.9%)	27 (26.73%)	2 (16.66%)	6 (37.5%)	0	
Low	63 (48.5%)	51 (50.49%)	6 (50%)	6 (37.5%)	0	
Type of ALL						
B-lymphocyte	65 (50%)	51 (50.49%)	5 (41.66%)	9 (59.25%)	0	0.137
T- lymphocyte	16 (12.3%)	10 (9.9%)	1 (8.33%)	4 (25%)	1 (100%)	

**Table 2:** Symptoms at diagnosis of acute lymphoblastic leukemia

Symptoms	Male, % (N=81)	Female, % (N=49)	Total, % (N=130)
Fever	27 (33)	17 (34.69)	44 (33.84)
Fatigue	10 (12.34)	4 (8.16)	14 (10.76)
Bone pain	5 (6.17)	7 (14.28)	12 (9.23)
Pale skin	9 (11.11)	2 (4.08)	11 (4.46)
Weight loss	6 (7.4)	3 (6.122)	9 (6.92)
Loss of appetite	6 (7.4)	3 (6.122)	9 (6.92)
Night sweat	3 (3.7)	2 (4.08)	5 (3.84)
Headache	2 (1.46)	1 (2.04)	3 (2.3)
Vomiting	1 (1.23)	1 (2.04)	2 (1.5)

Our data showed that 13.1% of our population had mutations. In addition, the highest frequency of mutations occurred in children (50.49%). No significant differences were observed between sex and age regarding mutations. Regarding nationality, 80.8% of the patients were non-Saudi. The mutations were significantly different according to nationality (p=0.042). Several gene mutations, including BCR-ABL and ETV6/RUNX1 were more common in B-ALL, whereas MLL-F0X04 and SIL/TAL1 were more common in T-ALL. The gene mutations were significantly different between the types of ALL (p=0.039) (Table 3). 

**Table 3:** Genes involved and types of ALL ALL, acute lymphoblastic leukemia

	BCR-ABL	ETV6/RUNX1	MLL-F0X04	p-value
B-ALL	11	4	0	0.039
T-ALL	1	0	1	
Children	8	3	1	0.862
Adolescents	2	1	0	
Adults	2	0	0	

BCR-ABL occurred in 9.2% (12/130) of patients; 7.92% (8/101) were found in children, 16.66% (2/12) in adolescents, and 12.5% (2/16) in adults. Four patients with B-ALL had the ETV6/RUNX1 mutation. One female patient had translocation t(X;11)(q26;q23) (MLL-F0X04). 

Ninety-two patients (70.8%) were treated with chemotherapy, 36 (27.7%) experienced relapse, and the remaining 38 patients (29.2%) required only best supportive care. At the end of the study period, 20.8% of the patients had died. No significant difference was observed between those who received chemotherapy and those who had relapse.

## Discussion

Mutations with acute lymphoblastic leukemia (ALL) have not been extensively investigated in Saudi Arabia. Therefore, we performed a retrospective study on patients diagnosed with ALL at the KAUH in Saudi Arabia to identify the types of gene mutations as well as the most common types of mutations in these patients. Among the 130 included patients, 13.1% had mutations; these mutations occurred more frequently in patients with B-ALL, accounting for 23% of these patients. Similarly, a previous study showed that more than two-thirds of B-ALL cases harbored harmful mutations targeting transcription [9].

Patients with B-ALL have a better prognosis than those with T-ALL, which is a more aggressive malignancy [4]. Several mutations, including BCR-ABL and ETV6/RUNX1, were more common in B-ALL; whereas STEL-TAL1 and MLL-F0X04 mutations were more frequent in T-ALL. This finding is consistent with those of a previous study [13]. BCR-ABL1 was the most common mutation in our population of patients. All cases of BCR-ABL were associated with B-ALL, except for one case that was associated with T-ALL; this finding is similar to that reported by Peking University First Hospital and Hebei Yanda Lu Daopei Hospital [13].

We observed that the majority of our population consisted of children (77.7%). This is consistent with other studies which showed that ALL was most common in children [5,6]. In relation to the mutations, a previous study demonstrated a positive relationship between age and BCR-ABL, reflecting that an increase in age will lead to an increase in the incidence of the mutation [10]. However, in our study, we found that BCR-ABL was more frequent in children than in the other age groups, representing 66.66% (8/11) of the cases. This finding might be due to unequal distribution in the age groups because children comprised more than half of our study population.

In a previous study of patients with ALL, the distribution of the ETV6/RUNX1 mutation increased around the age of one to 19 years [13]. In our study, four patients aged one, four, 10, and 19 years had this mutation. Similar to another study, we found that ETV6/RUNX1 was associated with B-ALL [12].

One female patient in our study had the translocation t(X;11)(q26;q23) (MLL-F0X04), which is a rare mutation; she was diagnosed with mixed-phenotype acute lymphoblastic and myeloblastic leukemia. The Tata Medical Center, Kolkata, reported that 2.2% of 628 patients with ALL had the MLL-F0X04 mutation [14]; our case was a 14-year-old female patient who presented with slight leukopenia (WBC count, 3.85 × 109/L) with a normal platelet count and manual differential counts showing increased neutrophils (72.6%), normal monocytic cells (7.5%), and low eosinophils (0.7%). However, the patient died a year after diagnosis.

Our results revealed that mutations in patients with ALL varied significantly. The frequencies of occurrence of these mutations varied among different nationalities, suggesting that these mutations are unstable.

Forty-two patients experienced relapse, and the lowest frequency of relapse was seen in children between two and 14 years of age (29.7%). The frequency of relapse was 41.66% in adolescents and 43.75% in adults. This result is consistent with that of a previous study in which 25% of children had relapse [7].

In addition, our patients with the ETV6/RUNX1 mutation did not experience relapse, a result similar to that of a study conducted in China, where they observed a low relapse rate in patients with ETV6/RUNX1. Moreover, in these patients, relapse occurred late, approximately 42 months after diagnosis [15].

Limitations

There were several limitations to our study. First, it was limited to a single center. Second, the relatively small study population and non-normal age distribution could have resulted in bias. We recommend performing the same study using a retrospective cohort design with a large population.

## Conclusions

In summary, 13.1% of our population had mutations, occurring frequently in patients with B-ALL. B-ALL harbored several mutations, including BCR-ABL and ETV6/RUNX1, whereas T-ALL harbored STEL-TAL1 and MLL-F0X04 mutations more frequently.

The BCR-ABL fusion gene is the most frequent mutation in patients at KAUH, and the BCR-ABL mutation occurs at a higher rate in B-ALL than in T-ALL. Moreover, we detected other mutations such as ETV6/RUNX1 and MLL-F0X04. The gene mutations were significantly different between B-ALL and T-ALL.
